# Dietary bile acids supplementation protects against *Salmonella* Typhimurium infection via improving intestinal mucosal barrier and gut microbiota composition in broilers

**DOI:** 10.1186/s40104-024-01113-5

**Published:** 2024-11-12

**Authors:** Dan Hu, Xiaoran Yang, Ming Qin, Li’an Pan, Haiyan Fang, Pengnan Chen, Yingdong Ni

**Affiliations:** Key Laboratory of Animal Physiology & Biochemistry, Nanjing Agricultural University, Nanjing, 210095 China

**Keywords:** Bile acid, Broiler, Intestinal barrier, Gut microbiota, *Salmonella* Typhimurium

## Abstract

**Background:**

*Salmonella* Typhimurium (*S*. Typhimurium) is a common pathogenic microorganism and poses a threat to the efficiency of poultry farms. As signaling molecules regulating the interaction between the host and gut microbiota, bile acids (BAs) play a protective role in maintaining gut homeostasis. However, the antibacterial effect of BAs on *Salmonella* infection in broilers has remained unexplored. Therefore, the aim of this study was to investigate the potential role of feeding BAs in protecting against *S*. Typhimurium infection in broilers.

**Methods:**

A total of 144 1-day-old Arbor Acres male broilers were randomly assigned to 4 groups, including non-challenged birds fed a basal diet (CON), *S*. Typhimurium-challenged birds (ST), *S*. Typhimurium-challenged birds treated with 0.15 g/kg antibiotic after infection (ST-ANT), and *S*. Typhimurium-challenged birds fed a basal diet supplemented with 350 mg/kg of BAs (ST-BA).

**Results:**

BAs supplementation ameliorated weight loss induced by *S*. Typhimurium infection and reduced the colonization of *Salmonella* in the liver and small intestine in broilers (*P* < 0.05). Compared to the ST group, broilers in ST-BA group had a higher ileal mucosal thickness and villus height, and BAs also ameliorated the increase of diamine oxidase (DAO) level in serum (*P* < 0.05). It was observed that the mucus layer thickness and the number of villous and cryptic goblet cells (GCs) were increased in the ST-BA group, consistent with the upregulation of *MUC2* gene expression in the ileal mucosa (*P* < 0.05). Moreover, the mRNA expressions of Toll-like receptor 5 (*TLR5*), Toll-like receptor 4 (*TLR4*), and interleukin 1 beta (*IL1b*) were downregulated in the ileum by BAs treatment (*P* < 0.05). 16S rDNA sequencing analysis revealed that, compared to ST group, BAs ameliorated the decreases in Bacteroidota, Bacteroidaceae and *Bacteroides* abundances, which were negatively correlated with serum DAO activity, and the increases in Campylobacterota, Campylobacteraceae and *Campylobacter* abundances, which were negatively correlated with body weight but positively correlated with serum D-lactic acid (D-LA) levels (*P* < 0.05).

**Conclusions:**

Dietary BAs supplementation strengthens the intestinal mucosal barrier and reverses dysbiosis of gut microbiota, which eventually relieves the damage to the intestinal barrier and weight loss induced by *S.* Typhimurium infection in broilers.

**Supplementary Information:**

The online version contains supplementary material available at 10.1186/s40104-024-01113-5.

## Introduction

As one of the most prevalent pathogens in poultry, *Salmonella* Typhimurium (*S*. Typhimurium) infection induces a multitude of intestinal diseases, including inflammation, barrier dysfunction, and disruption of the intestinal microbiota, thereby affecting the growth performance of chickens [1, 2]. Apart from causing substantial economic losses in poultry production, *S*. Typhimurium can transmit via the food chain, such as contaminated poultry meat, and cause foodborne salmonellosis, so it poses a threat to global public health [3, 4]. In fact, *S*. Typhimurium is a major cause of foodborne disease outbreaks worldwide, resulting in enteric infections in humans [5, 6]. Over the past few decades, antibiotics have been extensively employed to control *Salmonella* invasion in animals. However, due to significant drawbacks such as increased bacterial resistance and the presence of antibiotic residues in animal products, many countries and regions have prohibited the use of antibiotics as feed additives. Hence, exploring biological strategies for controlling *S*. Typhimurium infection is of great interest for chicken industry and human health concerns.

Bile acids (BAs) are a class of amphipathic molecules that include primary bile acids (PBAs) and secondary bile acids (SBAs). PBAs are synthesized from cholesterol in the liver and converted into SBAs by gut microbiota [7]. It is well established that BAs play important roles in facilitating dietary fat absorption in small intestine and lipid metabolism in liver [8, 9]. BAs also circulate continuously through the enterohepatic circulation and undergo microbial transformation by microbiota; thus, they also have profound effects on intestinal health as well as the gut microbiota balance [10]. It has been reported that tauroursodeoxycholate, a conjugated PBAs, inhibited intestinal inflammation and barrier disruption in mice with non-alcoholic fatty liver disease [11]. In addition, several BAs or BA-derived metabolites were recently demonstrated to play a role in alleviating intestinal inflammation in different types of colitis in mouse models [12].

Currently, researches on BAs in poultry have focused mostly on improving growth performance, regulating lipid metabolism, and promoting liver health [13–15]. In addition, studies have shown that BAs can promote the excretion of mycotoxins such as T-2 toxin and aflatoxin B, thereby improving the stress resistance of broilers and alleviating oxidative damage to the liver [16, 17]. Bansal et al. [18] have found that a secondary bile acid from microbiota metabolism attenuates ileitis in chickens, indicating a potential role for BAs in ameliorating intestinal injury in poultry. Additionally, our previous research has showed that dietary bile acid compounds improved gut microbiota in broilers feed a high-fat diet, increase the abundance of beneficial bacteria such as *Lactobacillus*, and significantly enhanced growth performance [13]. These results also suggest the beneficial effect of BAs on intestinal health. Nevertheless, the regulatory effect of BAs on intestinal health in poultry is still limited, and potential alleviating effect BAs intake on protecting against *S.* Typhimurium infection remains unknown. Therefore, the aim of this study was to investigate whether dietary BAs supplementation can protect against *Salmonella* Typhimurium infection by improving intestinal damage and microbiota.

## Methods

### Animal ethics and bird husbandry management

The animal experiments were approved by the Institutional Animal Care and Use Committee of Nanjing Agricultural University according to the Guidelines on Ethical Treatment of Experimental Animals (2006) No. 398 set by the Ministry of Science and Technology (2006, Beijing, China) (IACUC approval number: NJAU.No20231128180). All broilers were raised in a room with a stable temperature at 32 to 34 °C for the first 3 d, after which the temperature was decreased at a rate of 2 to 3 °C per week until d 19 (approximately 27 to 29 °C) with light throughout the day. The animals had free access to water and feed during the experiment.

### Experimental design and diets

**Fig. 1:**
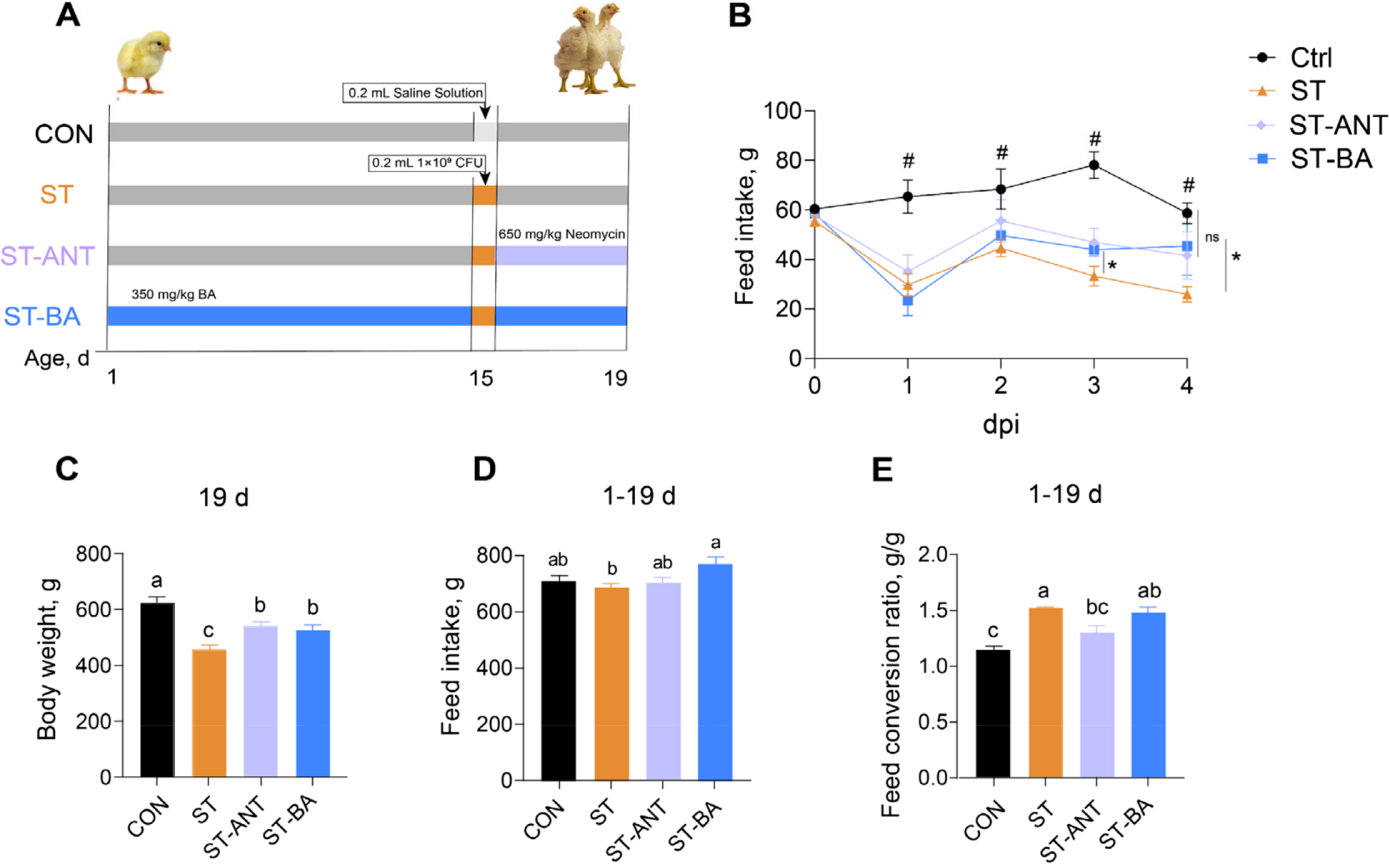
Experimental design and growth performance of broilers. Schematic diagram of the experimental design (**A**). The change of feed intake (FI) of broilers at 4 days post infection (dpi) (**B**). The body weight (BW) of broilers at d 19 (**C**). The feed intake of broilers from 1 to d 19 (**D**). The feed conversion ratio (FCR) of broilers from d 1 to 19 (**E**). *n* = 8 per group. The data are presented as means ± SEM. ^a^^–^^c^Different lowercase letters indicate that changes between groups are statistically significant. ^#^Significant difference between the CON group and ST group. *Significant difference between the ST group and the ST-BA group

A total of 144 1-day-old Arbor Acres male broilers were randomly assigned to 4 groups with six replicates and six birds per replicate. The 4 groups included birds fed a basal diet without *Salmonella* Typhimurium challenge (CON), *S*. Typhimurium-challenged birds (ST), *S*. Typhimurium-challenged birds treated with 0.15 g/kg antibiotic 24 h after infection (ST-ANT), and *S*. Typhimurium-challenged birds fed a diet supplemented with 350 mg/kg BAs (ST-BA). Intraperitoneal injection was used for *Salmonella* Typhimurium infection [19]. Except for those in the CON group, chicks in the other three groups received an intraperitoneal injection of *Salmonella* Typhimurium (1 × 10^9^ CFU) suspended in 0.2 mL of normal saline solution on d 15. Chicks in the ST-ANT group were treated with neomycin sulfate (Kedakang Animal Pharmaceutical Co., Ltd., Sichuan, China) added to drinking water at a concentration of 0.15 g/kg for 4 d started from 24 h after *S*. Typhimurium infection (Fig. 1A). Broilers were slaughtered at d 19, and samples were collected for the relevant analysis. The diet ingredients are shown in Table 1.

**Table 1:** The composition and nutritional levels of the basal diet

Ingredients	Content, %
Corn	55.63
Soybean meal	35.98
Soybean oil	3.80
Limestone	0.83
Calcium phosphate	1.74
Sodium chloride	0.36
L-Lysine	0.29
DL-Methionine	0.28
Threonine	0.09
Vitamin^1^	0.50
Trace mineral^2^	0.50
Total	100
Calculated analysis	
Crude protein, %	22.69
Available phosphorus, %	0.45
Calcium, %	1.04
Lysine, %	1.356
Digestible lysine, %	1.266
Methionine, %	0.597
Digestible Methionine, %	0.569
Crude fat, %	6.40
ME, kcal/kg	2,950

^1^Vitamins (per kg of diet): Vitamin A 15,000 IU, Vitamin D_3_ 3,600 IU, Vitamin E 50 IU, Vitamin K 4 mg, Vitamin B_1_ 3 mg, Vitamin B_2_ 10 mg, Vitamin B_5_ 10 mg, Vitamin B_6_ 3.6 mg, Vitamin B_12_ 0.03 mg, biotin 0.15 mg, nicotinamide 50 mg, pantothenic acid 12 mg, folic acid 1.5 mg

^2^Trace minerals (per kg of diet): Cu (copper) 10 mg, Fe (iron) 100 mg, Mn (manganese) 120 mg, Zn (zinc) 110 mg, I (iodine) 3.5 mg, Co (cobalt) 0.5 mg, Se (selenium) 0.3 mg,

### The composition of bile acids

The BAs were obtained from Shandong Longchang Animal Health Care Co., Ltd. (Dezhou, Shandong, China) and contained 70.67% hyodeoxycholic acid (HDCA), 19.61% chenodeoxycholic acid (CDCA), and 8.00% hyocholic acid (HCA) [15].

### Growth performance and organ weight

Broilers in each replicate were weighed on d 1 and 19. Body weight (BW) and daily feed intake were recorded to calculate weight gain (WG), feed intake (FI), and the feed conversion ratio (FCR). The weight of the liver, spleen and heart was recorded on d 19, and the organ index (organ weight [g]/body weight [kg]) was subsequently calculated.

### Sample collection

On d 19, after overnight fasting, 8 broilers from each group were randomly selected and slaughtered. Serum samples were collected and stored at −20 °C for further detection. After separated duodenum based on the location of the duodenal loop, the middle section of the proximal 2/3 (duodenal loop to Meckel’s diverticulum) of the remaining small intestine was collected as the sample for jejunal *Salmonella* counting. The tissue from the ileocecal folds was selected as the ileal sample, which distal section was used as the sample for ileal *Salmonella* counting. A segment of terminal-ileal tissue was rapidly removed and fixed with Carnoy’s fluid for hematoxylin-eosin (H&E) and Alcian blue/periodic acid-Schiff (AB/PAS) staining, and the remaining parts were frozen in liquid nitrogen immediately and stored at −80 °C for further analysis. The cecal digesta was collected and stored in sterile centrifuge tubes for 16S rDNA sequencing analysis.

### Detection of *Salmonella* Typhimurium colonization in tissues

Liver, jejunum and ileum tissues were sampled for bacterial load detection. The drop plate (DP) method was used to directly count viable *Salmonella* load in tissue as previously described [20]. Weigh 0.1 g tissue and then the tissue was homogenized and diluted 1:10 with sterile 0.85% sodium chloride. One hundred microliters of suspension were absorbed and diluted to 10^0^, 10^1^ and 10^2^. Five microliters were extracted from each concentration gradient and added to *Salmonella_Shigella* medium, with three replicates for each gradient. All plates were incubated overnight at 37 °C. The colony forming units per plate were counted, and then *S.* Typhimurium load in the tissues was calculated. The formula of calculating the *Salmonella* load is as follows:

$$Salmonella\;\text{load}\;\left(\text{CFU}/\text g\right)={{\text N}_{\text{c}}}\left(\text{CFU}\right)/\text{V}_\text{d}\left(\upmu \text{L}\right)\times1000\times \text{D}\times{\text{V}}_0\left(\text{mL}\right)/\text{M}\left(\text{g}\right)$$

where N_c_ is the number of colonies; V_d_ is the volume of droplets; D is the times of dilution; V_0_ is the volume of original solution; M is the mass of tissue.

### Serum diamine oxidase (DAO) activity and D-lactic acid (D-LA) concentration

The activity of serum DAO was detected using a commercial assay kit (Elabscience Biotechnology Co., Ltd., Wuhan, China). The serum concentration of D-LA was detected using an assay kit (Enzyme Linked Biotechnology Co., Ltd., Shanghai, China) following the manufacturer’s instructions.

### Ileum segment stained with hematoxylin-eosin (H&E) and Alcian blue/periodic acid-Schiff (AB/PAS)

For histological structure analysis, a segment of ileum tissue was fixed in Carnoy’s fluid, dehydrated and embedded in paraffin (*n* = 4/group). For H&E staining, six viewing fields (10× magnification) were selected for each sample to measure villus height and crypt depth and evaluate intestinal injury using histopathological scores according to previously described methods [21]. Briefly, three independent parameters measured were severity of inflammation (0–3: none, slight, moderate, severe), extent of injury (0–3: none, mucosal, mucosal and submucosal, transmural), and crypt damage (0–4: none, basal one-third damaged, basal two-thirds damaged, only surface epithelium intact, entire crypt and epithelium lost). The score of each parameter was multiplied by a factor reflecting the percentage of tissue involvement (×1, 0–25%; ×2, 26%–50%; ×3, 51%–75%; ×4, 76%–100%), and all numbers were summed. For AB/PAS staining, 1 to 3 fields of view under 20× magnification were selected to measure the mucus layer thickness, and the goblet cells were counted as previously described [22].

### Determination of gene expression using real-time quantitative polymerase chain reaction (RT-PCR)

Total RNA was isolated from ileum tissues using TRIzol reagent (TSP401, Tsingke, China). The concentration and quality of the RNA were measured with a NanoDropND1000 Spectrophotometer (Thermo Fisher Scientific, Madison, WI, USA). RNA samples were reverse transcribed with ABScript III RT Master Mix (AW311-02, TransGen, Beijing, China). A Genious 2X^®^ SYBR Green Fast qPCR Mix (RK21206, TransGen, Beijing, China) was used for real-time PCR, and β-actin was served as the reference gene. All primers were synthesized by Tsingke Company (Nanjing, China), and they are listed in Table 2. The data were analyzed using the 2^−∆∆Ct^ method, and the values were presented as the fold change compared to the CON group.

**Table 2:** Nucleotide sequences of specific primers

Target genes^1^	Sequences (5′→3′)	GenBank number
*MUC2*	GACAGCCAAGCAGGCAAGTG	XM_040673077.2
	GGTGGTGACATACTGCCAGA	
*Occludin*	AGGTCTGCAACAGCATCACA	NM_205128.1
	ATGCCTTCCCAAAAAGCCCT	
*ZO1*	GACAGCCAACAAGGCAAGTG	XM_046925214.1
	TGCATCCCATCCAGGTCCTA	
*CLDN1*	GGTATGGCAACAGAGTGGCT	NM_001013611.2
	CAGCCAATGAAGAGGGCTGA	
*JAM2*	ACTTGGGGGTCTTCTGCTATC	NM_001397141.1
	TACGGATTCCCGTATTCAGCA	
*TLR4*	GGCAGCTGACATCAGTCCTT	NM_001030693.2
	CCAGCTTCCAAGCACCAAAC	
*TLR5*	CTGTGTGTACCCACCTGCAT	NM_001398059.1
	AAAACAAATGCCCCGACAGC	
*MYD88*	AGGGATGATCCGTATGGGCA	NM_001030962.5
	AGCCCCTGGGGAAAGACTAA	
*IL1b*	GCCTGCAGAAGAAGCCTCG	NM_204524.2
	GGAAGGTGACGGGCTCAAAA	
*IL6*	AGGGCCGTTCGCTATTTGAA	NM_204628.2
	CAGAGGATTGTGCCCGAACT	
*IL8*	AGATGTGAAGCTGACGCCAA	NM_205018.2
	GAGCTGAGCCTTGGCCATAA	
*β-actin*	TGCTGTGTTCCCATCTATCG	L08165.1
	TTGGTGACAATACCGTGTTCA	

^1^*MUC2 *Mucin 2, *ZO1 *Zonula occludens 1, *CLDN1 *Claudin-1, *JAM2 *Junctional adhesion molecule 2, *TLR4 *Toll-like receptor 4, *TLR5 *Toll-like receptor 5, *MYD88 *Myeloid differentiation factor 88, *IL1b *Interleukin 1 beta, *IL6 *Interleukin 6, *IL8 *Interleukin 8

### DNA extraction and 16S rDNA sequencing

Cecal digesta samples were collected from broilers sacrificed on d 19. Six replicates per group were randomly chosen to characterize and quantitatively analyze the cecal microbiome by 16S rDNA sequencing. Microbial genomic DNA was extracted from cecal contents using a TGuide S96 Magnetic Stool DNA Kit (DP812, TIANGEN, Beijing, China). The concentrations of DNA in extracts were measured by microplate reader (GeneCompang Limited, USA). 16S rDNA was amplified using 338 F (5′-ACTCCTACGGGAGGCAGCA-3′) and 806R (5′-GTGYCAGCMGCCGCGGTAA-3′) primers through targeting the hypervariable V3–V4 regions, and 1.8% agarose gel electrophoresis was used to determine the concentration and purity of the DNA. Purified amplicons were sequenced on an Illumina NovaSeq 6000 platform (Illumina, San Diego, CA, USA), consistent with the standard protocols of Biomarker Technologies Co., Ltd. (Beijing, China). The raw data of sequencing has been uploaded to NCBI (http://www.ncbi.nlm.nih.gov/bioproject/1154557).

### 16S rDNA sequence processing and analysis

Raw reads were filtered by Trimmomatic (v0.33) [23]. Primer sequences were identified and removed by Cutadapt (v1.9.1) and obtained clean reads [24]. The clean reads of each sample were spliced through overlap using USEARCH (v10) and then chimera sequences were removed to obtain effective reads by using the UCHIME (v8.1) [25, 26]. Sequences were clustered into operational taxonomic units (OTUs) at a similarity level of 97% using USEARCH (v10), and OTUs were filtered with a threshold of 0.005% of all sequences [25]. Species annotation of OTUs was performed using bayesian classifier in QIIME2 (v2020.6), based on the Silva database (Release 138, http://www.arb-silva.de), with a classification confidence of 70% [27]. Venn diagram was created using R software (v3.1.1). Alpha diversity analysis was conducted by QIIME2 (v2020.6) and displayed by R software (v3.1.1). Beta diversity was evaluated by supervised partialleast squares discriminant analysis (PLS-DA) using R software (v3.1.1), based on ANOISM test. Circo graph of microbiota composition was displayed by Circos software (v0.66-7). Linear discriminant analysis Effect Size (‌‌LEfSe) analysis was conducted by python2 to identify microbes with significant differences in abundance among groups. The analysis of species differences among groups was conducted using analysis of variance (ANOVA), and the Tukey-Kramer was used for post-hoc comparisons. The bubble graph was drawn by R software (v3.1.1). Correlation analysis of the abundances of microbiota with body weight, organ indices and blood biochemistry parameters were performed by Spearman’s correlation analysis using Origin 2021 (OriginLab, Northampton, MA, USA). The correlation analysis heatmap was drawn by Origin 2021 and network diagrams were generated using Cytoscape (v3.10.2.)

### Statistical analysis

All the data were analyzed using IBM SPSS Statistics 27 (IBM, Armonk, NY, USA). One-way analysis of variance (ANOVA) was performed for comparisons of more than two groups, followed by Tukey’s test to determine the differences among groups. *P* < 0.05 indicated statistical significance. Other special analyses are described in the legends.

## Results

### Growth performance of broilers

The FI of the ST group broilers decreased continuously within 4 d after *S.* Typhimurium infection, and the broilers had a lower BW on d 19 and a higher FCR than broilers in the CON group (*P* < 0.05) (Fig. 1B and D). However, compared with *S.* Typhimurium-challenged broilers, dietary supplementation with BAs or antibiotic treatment significantly improved BW on d 19 and increased FI on the 3^rd^ and 4^th^ days after infection (*P* < 0.05) (Fig. 1B and C). Moreover, the FI of the ST-BA group was significantly increased from d 1 to 19 compared to that of the ST group (*P* < 0.05) (Fig. 1D). However, BAs supplementation had no influence on improving FCR (*P* > 0.05) (Fig. 1E). The data table is showed in Additional file 1.

### Organ weight and index of broilers

**Fig. 2:**
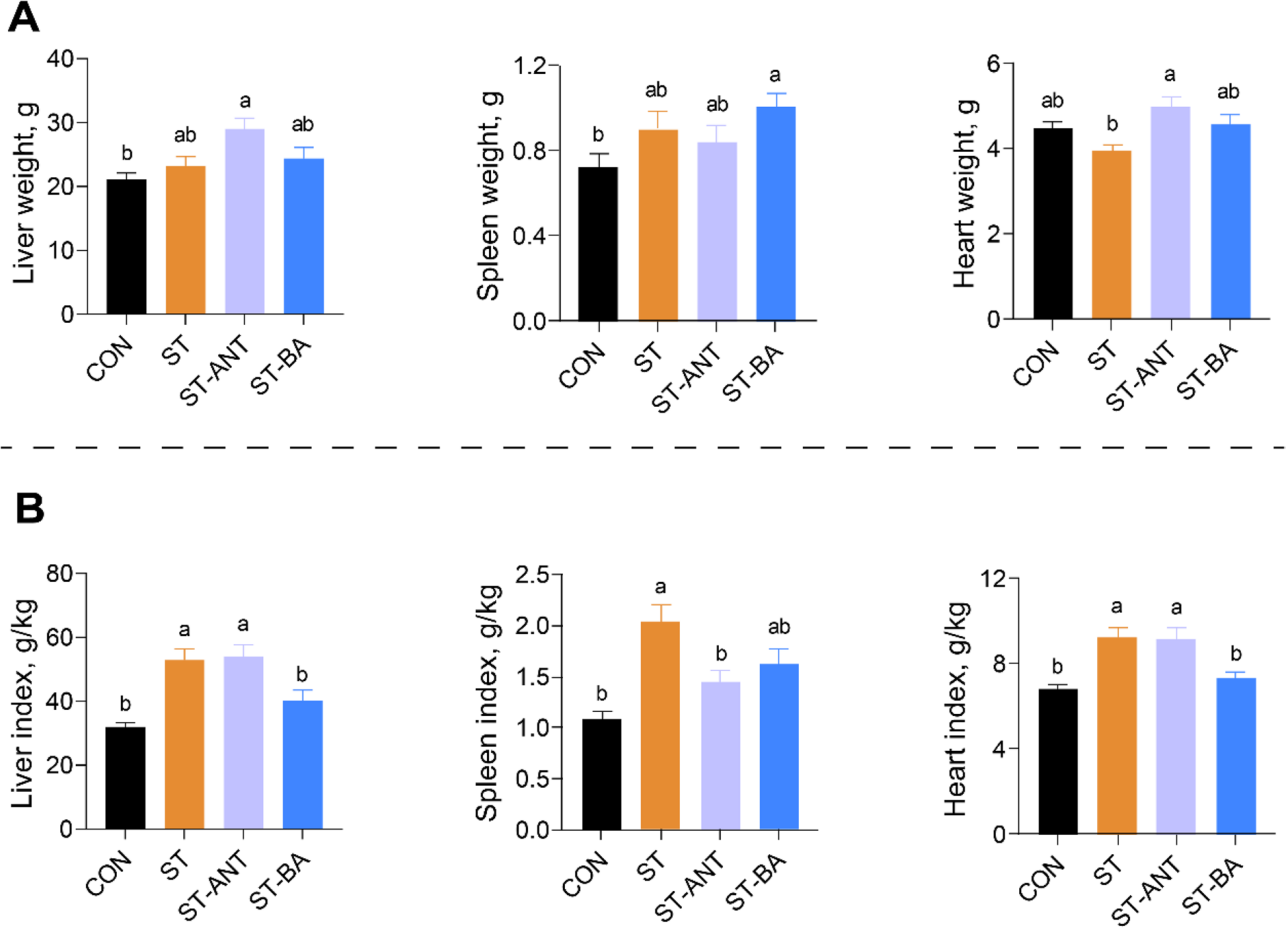
The weight and relative weight of the organs of the broilers. The weight of the liver, spleen, and heart (**A**) and the relative weight to body weight of liver, spleen, and heart (**B**). *n* = 8 per group. The data are presented as the means ± SEM. ^a,b^Different lowercase letters indicate that changes between groups are statistically significant

*S.* Typhimurium infection significantly increased the relative weight of the organs, including the liver, spleen and heart in broilers (*P* < 0.05). However, BAs supplementation significantly alleviated the increase in the liver and heart indices caused by *Salmonella* infection (*P* < 0.05), while the administration of antibiotics significantly reduced the spleen index (*P* < 0.05) (Fig. 2 and Additional file 1).

### Colonization of *Salmonella* in the tissues of broilers

**Fig. 3:**
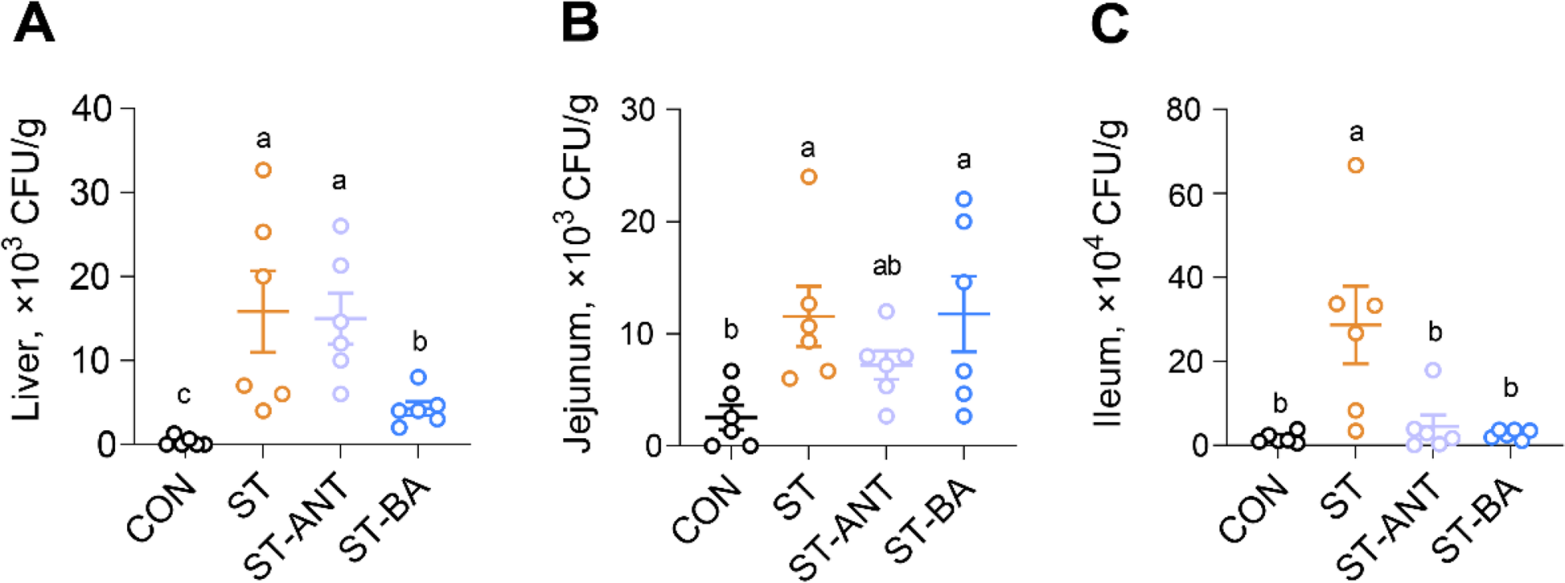
The load of *Salmonella* in the tissues of broilers. The load of *Salmonella* in liver (**A**), jejunum (**B)** and ileum (**C**). *n* = 6 per group. The data are presented as the means ± SEM. ^a–c^Different lowercase letters indicate that changes between groups are statistically significant

The bacterial load in organs after *Salmonella* infection was evaluated by viable plate count. Results showed that *Salmonella* colonization in the liver reached 10^4^ CFU/g in ST group, which was significantly higher than that in the CON group (*P* < 0.05) (Fig. 3A), and a higher load of *Salmonella* was detected in the jejunum and ileum tissues of *S.* Typhimurium-challenged broilers (*P* < 0.05) (Fig. 3B and C). Dietary supplementation with BAs significantly reduced the colonization of *Salmonella* in the liver and ileum (*P* < 0.05), while antibiotic treatment decreased bacterial colonization in the ileum (*P* < 0.05) (Fig. 3A and C).

### Small intestinal development and morphology of broilers

**Fig. 4:**
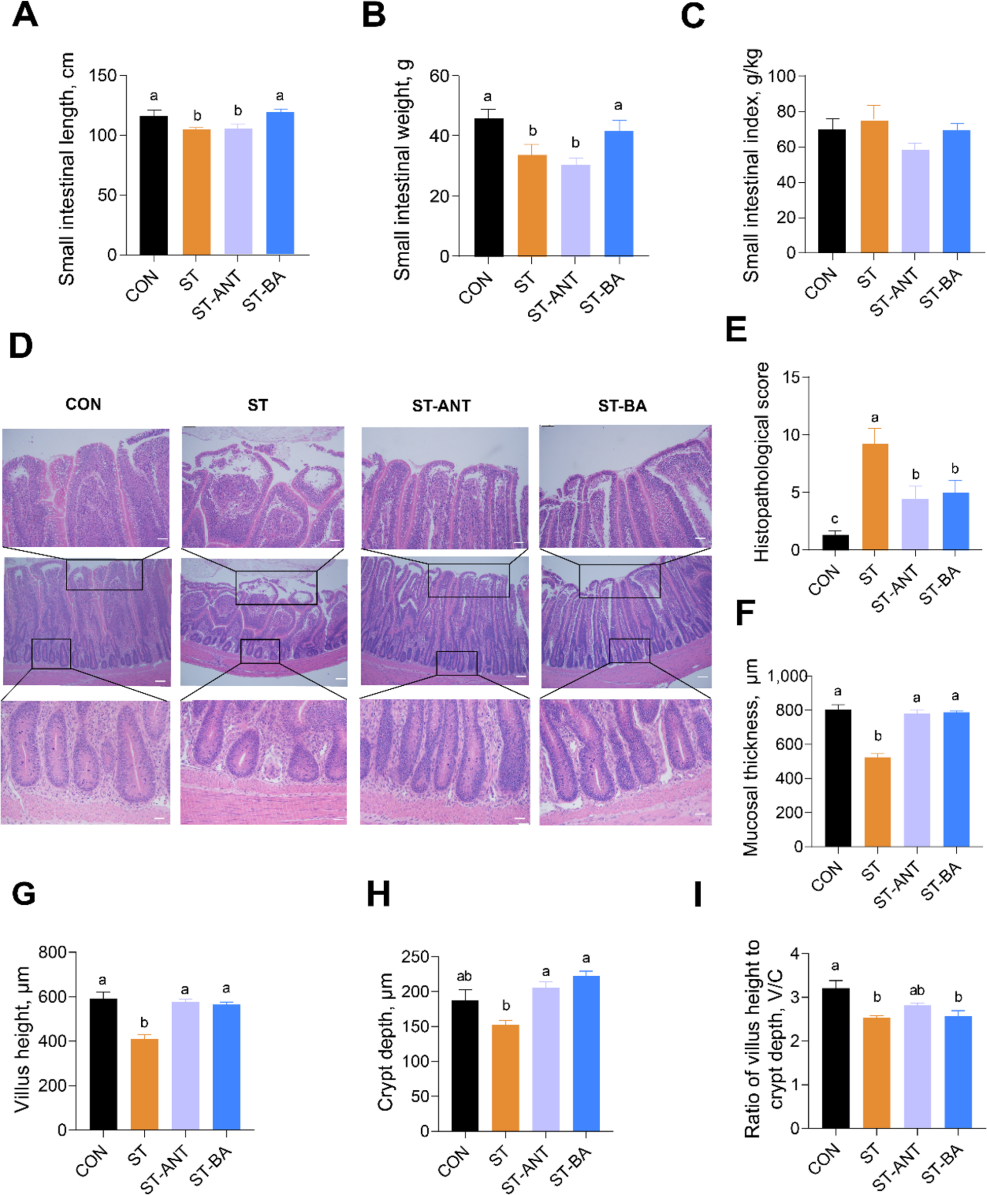
Small intestinal development and morphology of broilers. The length (**A**), weight (**B**) and index (**C**) of the small intestine in broilers; *n* = 8 per group. H&E staining (**D**) and histopathological score of the ileum (**E**). Statistics of mucosal thickness (**F**), villus length (**G**), crypt depth (**H**) and the ratio of villus height to crypt depth (V/C) (**I**) in the ileum; *n* = 4 per group. ^a–c^The data are presented as the means ± SEM. Different lowercase letters indicate that changes between groups are statistically significant

As shown in Fig. 4, compared with broilers in the CON group, the length and weight of small intestine were significantly decreased in the ST and ST-ANT groups (*P* < 0.05) (Fig. 4A–C). However, the length and weight of the small intestine in the ST-BA group were significantly increased compared to the ST group (*P* < 0.05) (Fig. 4A and B). H&E staining of the ileal epithelium also detected an orderly arrangement of villi and an intact structure of crypts, as observed in the CON group. In contrast, the *S*. Typhimurium-challenged group showed evident villus shedding, loss of crypts and a higher histopathological score (*P* < 0.05) (Fig. 4D and E). Consistently, the statistical results revealed a significant reduction in intestinal mucosal layer thickness in the ST group, accompanied by a decrease in villus height and the ratio of villus height to crypt depth (V/C) (*P* < 0.05) (Fig. 4F–I). Although the ST-ANT and ST-BA groups showed slight villus damage, both intestinal mucosal thickness and villus height were higher than those in the ST group and had lower damage scores (*P* < 0.05) (Fig. 4D–I).

### Intestinal barrier permeability and mucus barrier

**Fig. 5:**
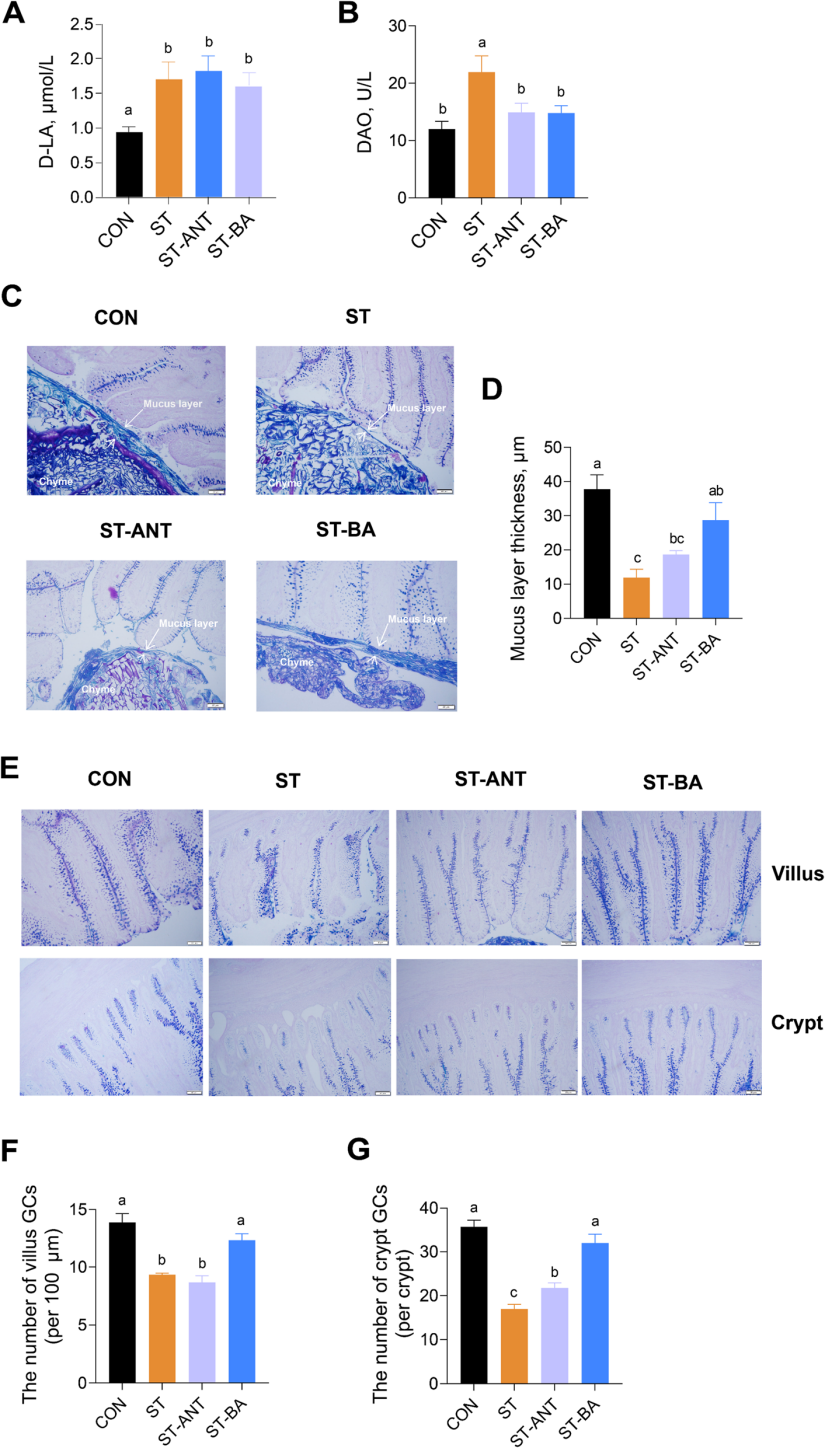
Intestinal barrier permeability and mucus barrier integrity in the ileum of broilers. D-Lactic acid (D-LA) concentration (**A**) and diamine oxidase activity (DAO) (**B**) in serum; *n* = 8 per group. Representative Alcian blue/periodic acid-Schiff (AB/PAS) images showing mucus layer thickness and the number of goblet cells (**C**–**G**); *n* = 4 per group. The data are presented as the means ± SEM. ^a–c^Different lowercase letters indicate that changes between groups are statistically significant

The results showed that the levels of DAO and D-LA in the ST group were higher than the CON group, while the DAO levels in both the ST-BA and ST-ANT groups were decreased compared to the ST group (*P* < 0.05) (Fig. 5A and B). *S. *Typhimurium infection significantly reduced the thickness of the mucus layer (*P* < 0.05) (Fig. 5C and D). Similarly, the number of GCs in villi and crypts, which secrete mucin 2, the main component of mucus, was significantly reduced in the ST group compared to the CON group (*P* < 0.05) (Fig. 5E–G). However, the mucus layer thickness and the number of GCs in both villi and crypts were significantly increased by BAs supplementation but not antibiotic treatment (*P* < 0.05).

### Expression of barrier-related and inflammation-related genes in the ileum

**Fig. 6:**
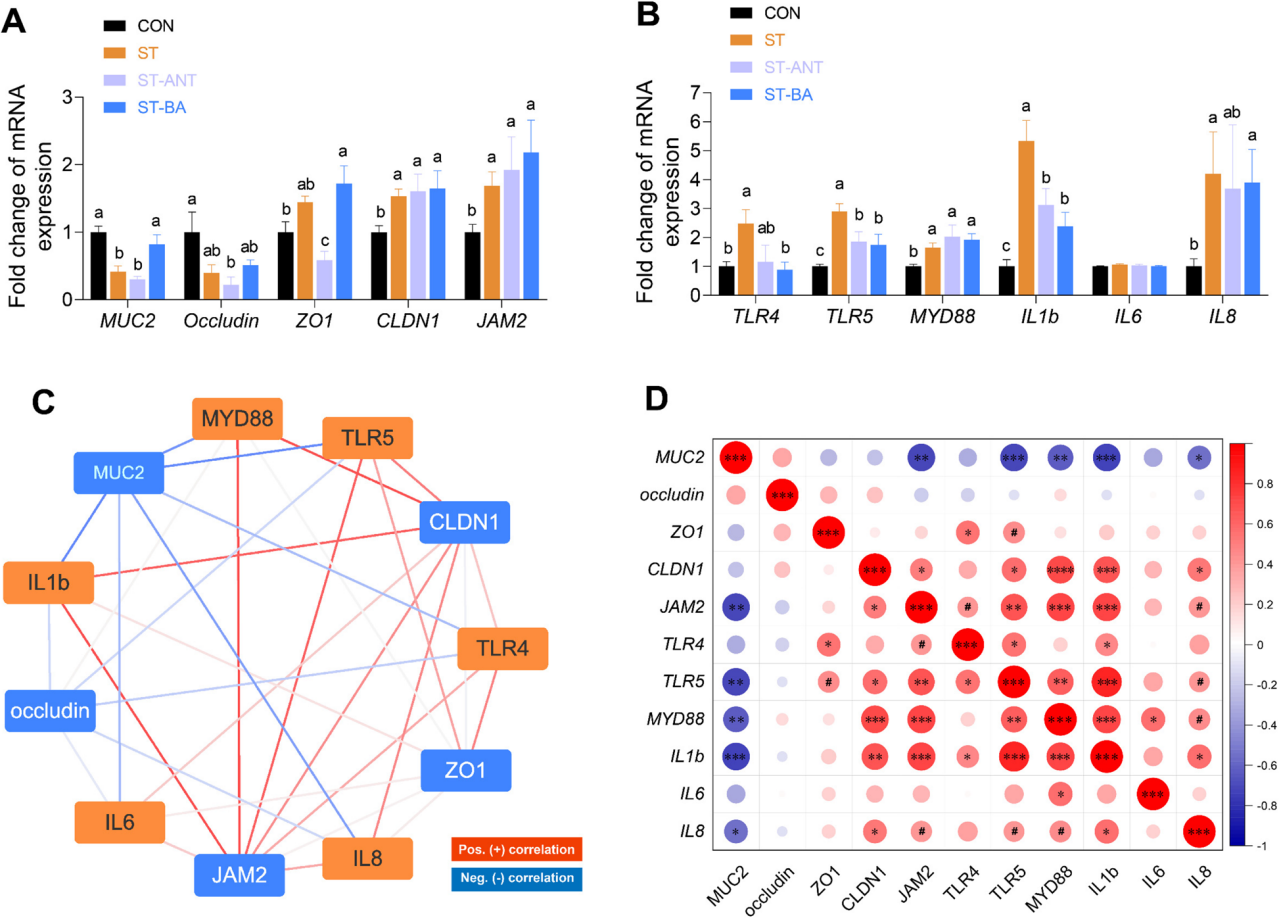
Expression of genes related to the ileal barrier and inflammation. The relative expression of barrier-related genes (**A**). The relative expression of inflammation-related genes (**B**). Spearman correlation analysis based on the network diagram (**C**) and the heatmap (**D**) at gene expression level (|*R*| > 0.6, *P* < 0.05). *MUC2*, mucin 2; *ZO1*, zonula occludens 1; *CLDN1*, claudin-1; *JAM2*, junctional adhesion molecule 2; *TLR4*, Toll-like receptor 4; *TLR5*, Toll-like receptor 5; *MYD88*, myeloid differentiation factor 88; *IL1b*, interleukin 1 beta; *IL6*, interleukin 6; *IL8*, interleukin 8. The data are presented as the means ± SEM of 8 replicates in each group (*n* = 8). ^a–c^Different lowercase letters indicate that changes between groups are statistically significant. ^#^*P* < 0.1, ^*^P < 0.05, ^**^P < 0.01, ^***^P < 0.001

*S.* Typhimurium infection reduced the mRNA expression of mucin 2 (*MUC2*) in the ileum, although it increased the expression of claudin-1 (*CLDN1*) and junctional adhesion molecule 2 (*JAM2*) (*P* < 0.05; Fig. 6A). However, the decrease in *MUC2* expression was reversed by BAs treatment. As Gram-negative bacteria, LPS and flagellin produced by *Salmonella* can activate Toll-like receptor 4 (TLR4) and Toll-like receptor 4 (TLR5), respectively. The mRNA expression of *TLR4* and *TLR5* in the ileal mucosa was significantly upregulated by *Salmonella* infection compared to the CON group (*P* < 0.05; Fig. 6B). Subsequently, the mRNA expression of downstream responsive proteins, including myeloid differentiation factor 88 (*MYD88*), pro-inflammatory cytokines interleukin 1 beta (*IL1b*) and *IL8*, also significantly increased following the activation of TLR4/5 (*P* < 0.05). However, significant downregulation of *TLR4*,* TLR5* and *IL1b* mRNA expression in the ileum was observed in both the ST-BA and ST-ANT groups compared to the ST group (*P* < 0.05). Correlation analysis revealed that *MUC2* expression was significantly negatively correlated with the inflammatory cytokines *TLR5*, *MYD88*, *IL1b*, and *IL8* (|*R*|> 0.6, *P* < 0.05; Fig. 6C and D). These findings further provided clues that BA could improve the intestinal mucus layer structure as well as mitigating inflammatory responses and disease progression via mucus production during *Salmonella* infection.

### Analysis of the cecal microbiota community by 16S rDNA sequencing

#### Diversity and composition of microbiota

**Fig. 7:**
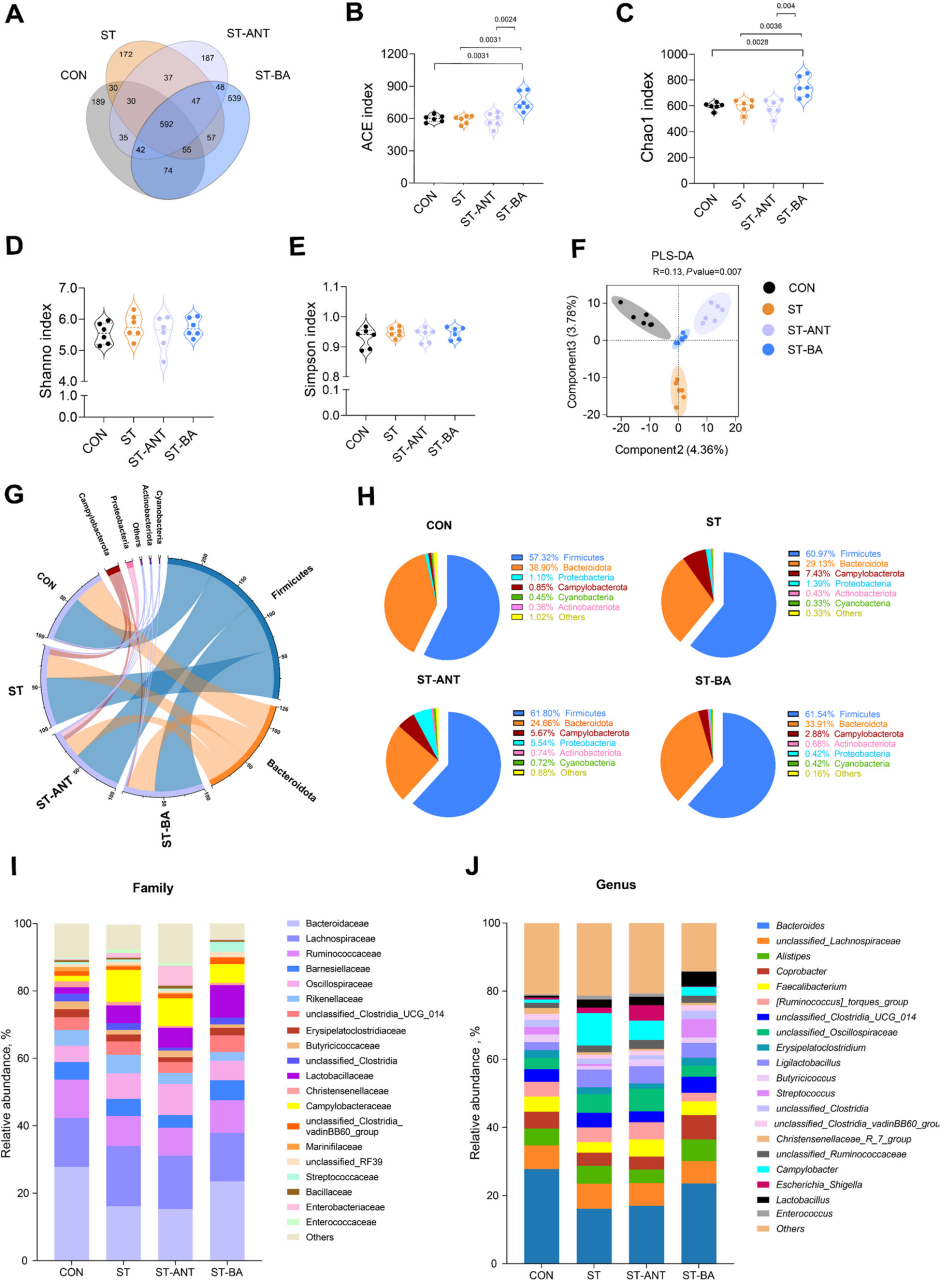
Microbiota composition and diversity in cecum content of broilers. Venn diagram showing the number of operational taxonomic units (OTUs) (**A**). Alpha diversity including ACE (**B**), Chao1 (**C**), Shannon (**D**) and Simpson (**E**) diversity index on OTUs. Beta diversity analysis based on PLS-DA analysis on OTUs (**F**). Circos (**G**) and pie (**H**) graphs of microbiota composition in phylum level. Relative abundance of bacteria at the family (**I**) and genus level (**J**). *n* = 6 per group. The data are presented as means ± SEM

The gut microbiota in the cecal contents of broilers was characterized and quantitatively analyzed via 16S rDNA sequencing. Venn diagram showed that there were 1,047 OTUs in the CON group, 1,020 in the ST group, 1,080 in the ST-ANT group and 1,434 in the ST-BA group (Fig. 7A). Additionally, α-diversity analysis showed higher Chao1 and ACE indices in the ST-BA group compared to the other groups (*P* < 0.05), indicating increased richness of microbiota in the cecum (Fig. 7B–E). However, the Simpson and Shannon indices did not show significant differences among the groups (*P* > 0.05). Beta-analysis revealed structural differences in the gut microbiota among the groups (*P* < 0.05; Fig. 7F). The main phyla of microbiota in the cecal contents of broilers were Firmicutes and Bacteroidota. However, the abundance of Bacteroidota in the ST and ST-ANT groups (29.13% and 24.66%, respectively) was lower than CON and ST-BA groups (38.90% and 33.91%, respectively), while the abundance of Campylobacterota in the ST and ST-ANT groups (7.43% and 5.67%, respectively) was higher than CON and ST-BA groups (0.85% and 2.88%, respectively; Fig. 7G and H). Consistently, within Bacteroidota, the abundances of Bacteroidaceae and *Bacteroides* in the ST-BA group were higher than those in the ST group (*P* < 0.05; Fig. 7I and J).

#### Differential bacteria in the gut microbiota among groups

**Fig. 8:**
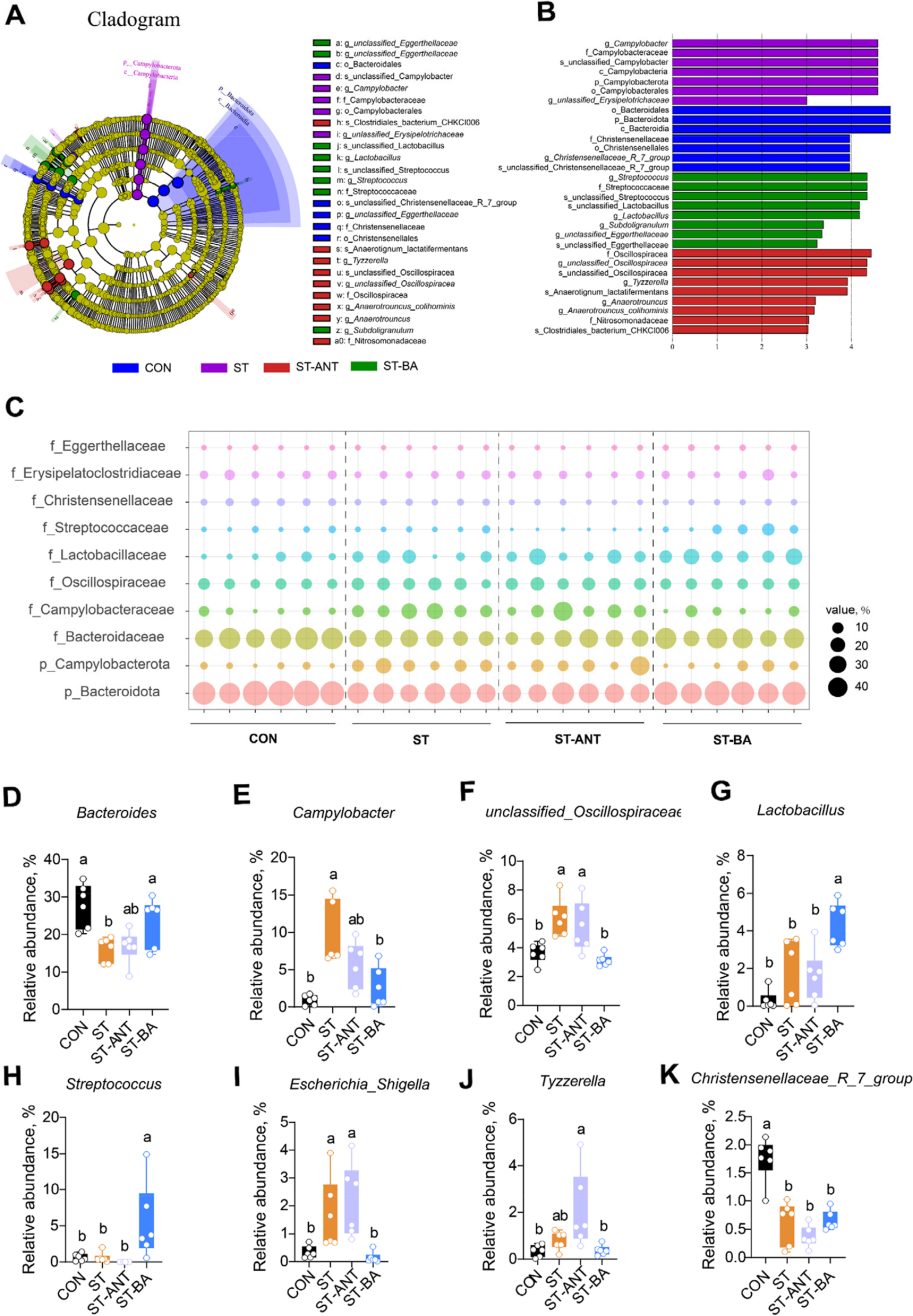
Differentially abundant bacteria in the gut microbiota among groups. The differentially abundant bacteria among the groups are presented in the LDA cladogram generated via LEfSe analysis (*P* < 0.05, LDA > 3.0) (**A** and **B**). Bubble diagram showing significantly different microbiota at the phylum and family levels (**C**). Significantly different microbiota at the genus level (**D**–**K**). *n* = 6 per group. The data are presented as the means ± SEM. ^a,b^Different lowercase letters indicate that changes between groups are statistically significant

As shown in Fig. 8, LEfSe analysis (*P* < 0.05, LDA > 3.0) revealed that certain bacterial members, such as the phylum Bacteroidota, class Bacteroidales, and order Bacteroidia, were enriched in the CON group. The phylum Campylobacterota, class Campylobacteria, order Campylobacterales, family Campylobacteraceae and genus *Campylobacter* were the dominant bacterial groups enriched in the ST group. The genus *Lactobacillus* and family Oscillospiraceae were mainly enriched in the ST-BA and ST-ANT groups, respectively. Consistent with the above results, compared to the CON group, the relative abundances of Bacteroidota, Bacteroidaceae, *Bacteroides*, Christensenellaceae, and *Christensenellaceae_R_7_group* were significantly decreased in the *S.* Typhimurium-challenged broilers, while the abundances of harmful bacteria, including Campylobacterota, Campylobacteraceae, *Campylobacter* and *Escherichia_Shigella*, were significantly increased. In contrast, BAs supplementation led to higher abundances of Bacteroidaceae and *Bacteroides* but lower abundances of Campylobacteraceae, *Campylobacter* and *Escherichia_Shigella* in cecum than ST-challenged group. Moreover, dietary supplementation with BAs significantly increased the abundance of the *Lactobacillus* and *Streptococcus* genera. However, antibiotic treatment promoted the abundance of Oscillospiraceae and *unclassified_Oscillospiraceae* in the cecal content of the broilers.

#### Correlation analysis

**Fig. 9:**
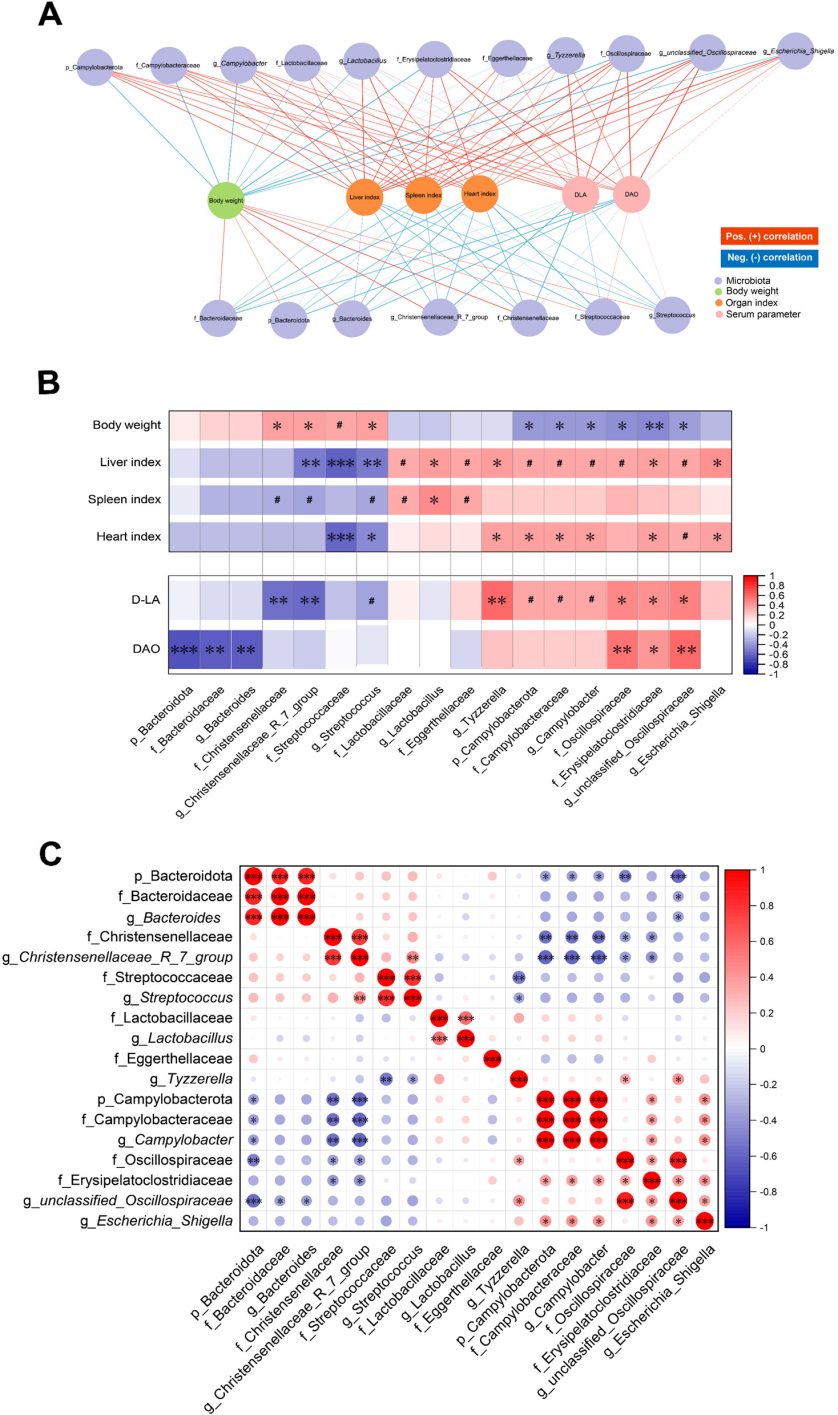
Correlation analysis. Spearman correlation analysis based on the network diagram (**A**) and the heatmap (**B**) between the microbiota and both growth performance and serum indices (|*R*| > 0.6, *P* < 0.05). Spearman correlation analysis based on a heatmap of the microbiota (|*R*| > 0.6, *P* < 0.05) (**C**). ^#^*P* < 0.1, ^*^P < 0.05, ^**^P < 0.01, ^***^P < 0.001

Subsequently, we conducted a correlation analysis between differential microbial communities and parameters, including body weight, the indices of liver, heart and spleen, as well as serum DAO and D-LA levels (Fig. 9A and B). The relative abundances of Christensenellaceae and *Christensenellaceae_R_7_group* were significantly lower in the ST group, exhibiting significant positive correlations with BW but negative correlations with the indices of the liver, spleen and heart, as well as blood D-LA level (|*R*| > 0.6, *P* < 0.05). The decreased abundances of the taxa Bacteroidota, Bacteroidaceae and *Bacteroides* also showed significant negative correlations with serum DAO level (|*R*| > 0.6, *P* < 0.05). Conversely, the relative abundances of Campylobacterota, Campylobacteraceae, and *Campylobacter* increased in the ST group, which were negatively correlated with body weight but positively correlated with liver index, heart index and serum D-LA level (|*R*| > 0.55, *P* < 0.1). A positive correlation was observed between *Streptococcus* abundance and BW, and a negative correlation was observed between *Streptococcus* abundance and the indices of the liver and heart (|*R*| > 0.6, *P* < 0.05). Additionally, *Streptococcus* was negatively correlated with the serum DAO level (|*R*| > 0.55, *P* < 0.1). However, the relative abundances of Oscillospiraceae and *unclassified_Oscillospiraceae* were negatively correlated with BW but were positively correlated with serum DAO and D-LA levels (|*R*| > 0.6, *P* < 0.05). Finally, considering the competitive and symbiotic relationships within the gut microbiota, we conducted a reciprocal correlation analysis of the microbial communities (Fig. 9C). *Campylobacter* abundance was negatively correlated with both Bacteroidaceae and Christensenellaceae at the family level, (|*R*| > 0.6, *P* < 0.05). Meanwhile, the positive correlation between *Escherichia_Shigella* and *Campylobacter* might reflect a symbiotic relationship (|*R*| > 0.6, *P* < 0.05). These results further demonstrated the importance of gut microbiota homeostasis in resisting *Salmonella* infection.

## Discussion

Salmonellosis is a bacterial disease susceptible to poultry and is characterized by intestinal damage, diarrhea, and systemic infection in severe cases, which eventually leads to retarded growth performance and even death [28, 29]. Currently, an effective strategy for treating *Salmonella* infections is still the use of antibiotic, such as neomycin, following the drawback of antibiotic resistance [30, 31]. In this research, we explored the role of BAs in protecting against *S.* Typhimurium infection, especially in terms of intestinal health.

Consistent with previous studies [19, 32], in the present study, *Salmonella* infection significantly reduced feed intake from d 16 to 19 and body weight on d 19. However, dietary supplementation with BAs or antibiotic treatment significantly improved BW on d 19 and increased FI on d 3 and 4 post-challenge. It should be noted that the decrease of FI in the control group on 4 dpi was due to overnight fasting before slaughter. A significant increase in the relative weight of the liver, spleen and heart to body weight indicated inflammatory edema of organs after *Salmonella* infection. However, dietary BAs supplementation can greatly decrease the relative weight of the liver and heart to normal levels, and antibiotic treatment significantly reduced the relative weight of the spleen. Extensive researches have demonstrated the significant growth-promoting effect of bile acids in poultry [13, 14, 33]. Actually, a significant increase in the body weight of broilers fed bile acids was observed on 14 d (pre-*Salmonella* infection) in this study (data not shown). It is reasonable to speculate that the improvement in body weight after infection might be mostly attributed to the “priming effect” of BAs, which differed from antibiotic treatment.

The counts of viable *Salmonella* colonized in the jejunum, ileum, and liver tissues were increased in the ST group, which was consistent with previous findings [34]. With potent antibacterial activity, experiments have shown that BAs can significantly inhibit the invasion of HeLa cells by *Salmonella* Typhimurium [35]. Recently, in vitro studies also demonstrated that CDCA, a primary BA, effectively inhibited the expression of virulence genes and epithelial cell invasion by *S.* Typhimurium, interacting with numerous proteins and thereby reducing its pathogenicity [36]. In this study, dietary BAs supplementation significantly decreased the load of *Salmonella* in the liver and ileum but not in the jejunum. This difference might be related to the sensitivity of tissues to bile acid signaling. The liver and ileum are crucial sites for the enterohepatic circulation of BAs, which exhibit high expression of bile acid receptors such as FXR and TGR5 [37, 38]. However, antibiotic treatment reduced the abundance of *Salmonella* in the intestine, suggesting that antibiotics might primarily play a role through direct bactericidal mechanisms.

The invasion of harmful bacteria into the intestinal epithelium can trigger an inflammatory response and disrupt the intestinal barrier, resulting in intestinal “leakage” and facilitating the longitudinal translocation of pathogenic bacteria and their metabolites from the intestinal lumen into the circulatory system [39, 40]. DAO is an enzyme in intestinal epithelial cells in humans and most species of animals, while D-LA is a bacterial metabolic product that cannot be decomposed by the enzymatic system of the host [41, 42]. Higher levels of DAO and D-LA in the blood reflected lower integrity of the intestinal epithelium barrier. In order to evaluate intestinal barrier permeability in broilers, the activity of DAO and the concentration of D-LA in the serum were measured. The serum levels of DAO and D-LA were significantly increased in the ST group, indicating intestinal mucosal barrier disruption. Consistently, histopathological examination also revealed fractured villus damage and thinner mucosal thickness in ST-challenged broilers compared to CON group. However, both antibiotic treatment and BAs supplementation reduced the serum DAO level and improved intestinal mucosal damage.

As a Gram-negative bacterium, *Salmonella* produces LPS that can activate intestinal epithelial TLR4 receptors, inducing innate immune responses [43]. Moreover, it also hijacks host signaling pathways via its secreted flagellin during infection, which directly binds to its receptor TLR5 on cells in mucosal tissues and then activates immune cells to induce the expression of pro-inflammatory cytokines [44]. We also detected significant upregulation of the gene expression of *TLR4* and *TLR5* in the intestine of *S*. Typhimurium-challenged broilers. Subsequently, the expression of genes encoding downstream ligands and inflammation-related cytokines, including *IL1b*, *IL6* and *IL8*, was also upregulated in the ileal epithelium in ST-infected chickens. As signaling molecules, BAs such as deoxycholic acid (DCA), HDCA and lithocholic acid (LCA) and their derivatives have been reported to activate the FXR or TGR5 signaling pathway or regulate immune cells migration and differentiation to improve inflammation [12, 45]. In this study, BAs decreased the expression of *TLR4*, *TLR5* and *IL1b* mRNA in the ileal mucosa, highlighting the role of BAs in protecting the intestinal barrier of broilers against *S*. Typhimurium invasion.

The mucus barrier and epithelial barrier are important components of the intestinal barrier [46, 47]. The mucus barrier, primarily composed of mucus and mucins secreted by GCs, provides the first line of defense that prevents direct contact between intestinal bacteria and epithelial cells [48]. Previous research on poultry has demonstrated that chickens infected with *Salmonella* exhibit a decrease in both the number of GCs and MUC2 expression in the intestine [49, 50]. Similarly, broilers in the ST group in this study showed a decrease in the number of GCs in both villi and crypts, along with a significant reduction in mucous layer thickness. Intestinal GCs originate from crypt stem cells and continuously migrate and differentiate toward villi [51]. Research in mice has revealed that BAs can activate intestinal stem cells via the TGR5 signaling pathway, promoting the differentiation of stem cells into various epithelial cell types, including GCs [52]. Furthermore, Song et al. [53] found that CDCA and DCA can induce MUC2 transcription in human colon carcinoma cells using MUC2 promoter reporter luciferase assay. DCA induced MUC2 expression in GCs cultured in vitro [54]. Our results also showed that BAs treatment significantly increased the number of GCs in the villi and crypts of the ileum, and the expression of *MUC2* significantly increased, restoring the loss of GCs and mucus in broilers suffering from *Salmonella* infection. In fact, the level of MUC2 expression and the number of GCs are closely related to the activation of the inflammatory response in the intestine [48]. Researches have demonstrated that MUC2-deficient mice were more susceptible to colitis, and the level of MUC2 expression was significantly negatively correlated with the expression of inflammatory cytokines [55, 56]. Our results also revealed a negative correlation between the expression of *MUC2* and inflammatory cytokines, especially *TLR5*, *MYD88* and *IL1b*. Therefore, it can be inferred that supplementation of BAs in broilers can also promote mucus secretion by increasing the number of goblet cells and the expression of MUC2, thus maintaining the thickness of the mucosal layer and protecting the intestinal epithelium from inflammatory lesions. In contrast to the effects of BAs, antibiotic treatment had no significant effect on the number of goblet cells or the expression of *MUC2*. However, regarding the intestinal epithelial barrier, contrary to other reports [50, 57], *Salmonella* infection significantly increased the expression of the tight junction-related genes *CLDN1* and *JAM2*. This might represent a compensatory response to mucus damage in the intestine.

The gut microbiota can act as a mediator in intestinal lesions triggered by *Salmonella*, exerting profound impacts on intestinal health and growth performance in chickens, particularly in the context of infection [58, 59]. Consistent with previous research [57], *Salmonella* infection did not alter the α diversity of the gut microbiota, but significant differences were observed in β diversity compared to the CON group, indicating a significant change in the composition of the gut microbiota. Research has indicated that BAs treatment elevated gut microbiota richness in poultry [13]. In this study, the ACE and Chao1 indices of the ST-BA group significantly increased. Moreover, β-diversity analysis revealed significant separation in microbial composition between the ST-BA group and the other three groups.

Thiam et al. [60] revealed that Bacteroidetes might be involved in resistance against *Salmonella* infection in chickens, and the abundance of Bacteroidetes decreased significantly in egg-laying chickens infected with *Salmonella* [61]. Similarly, we also observed a decrease in the abundance of Bacteroidota, Bacteroidaceae and *Bacteroides* in the ST group, and their abundance also showed significant negative correlations with the serum DAO level. In addition, our results showed a decrease in the abundance of Christensenellaceae and *Christensenellaceae_R_7_group *in broilers infected with *Salmonella*, which are considered as probiotics in humans [62]. Recently, microbiome analysis in chickens identified the cecal taxa Christensenellaceae and *Christensenellaceae_R_7_group* as representative taxa contributing to the combined effect of host genetics and short-chain fatty acids feed efficiency [63]. Notably, the relative abundances of Christensenellaceae and *Christensenellaceae_R_7_group* were positively correlated with BW but negatively correlated with the liver index, spleen index, heart index, and blood D-LA level in this study. Alternatively, the abundance of *Campylobacter *and* Escherichia_Shigella* and was increased in the ST group, and the abundance of *Campylobacter* was negatively correlated with body weight but positively correlated with liver index, heart index and serum D-LA level. Similarly to *Salmonella*, *Campylobacter* is also considered as one of a typical pathogenic bacterium in humans and animals, result in the culprits of diarrhea and foodborne illnesses, especially in chickens [6]. Besides, our result showed that *Campylobacter* abundance was negatively correlated with both Bacteroidaceae and Christensenellaceae, suggesting potential competitive relationships among these microorganisms.

BAs play an important role in shaping the microbial ecology of the gut. Extensive studies have indicated that bile acids can inhibit the growth of specific bacteria, such as *Escherichia coli* and *Bacillus* in vitro [64, 65]. Sun et al. [66] and Jiang et al. [67] have confirmed that oral administration of microbiota-derived secondary BAs inhibited *Campylobacter* infection in the gut of mice, while DCA can reduce the colonization of *Campylobacter* in the jejunum and increase the abundance of *Bacteroides* [68]. Similarly, this research also demonstrated that the addition of bile acids improved the decrease in *Bacteroides* and the increase in *Campylobacter* and *Escherichia_Shigella* in *S.* Typhimurium-challenged broilers. Moreover, consistent with previous reports [13], BAs supplementation increased the abundance of *Lactobacillus* and *Streptococcus*, and the abundance of *Streptococcus* was positively correlated with BW but negatively correlated with liver and heart indices. It can be argued that microbiota dysbiosis driven by *Salmonella* infection may further exacerbate intestinal lesions and even cause other harmful bacterial contamination, while BAs can ameliorate the detrimental effects and increase the abundance of beneficial bacteria.

## Conclusion

Dietary supplementation with BAs reduced *Salmonella* colonization in tissues of broilers. BAs strengthen the intestinal mucosal barrier by increasing the number of goblet cells and promoting MUC2 expression, and BAs also improved dysbiosis of the gut microbiota, thereby alleviating intestinal lesions and growth retardation in *S.* Typhimurium-challenged broilers.

## Supplementary Information

**Additional file 1. **Growth performance and the weight of organs of broilers.

## Data Availability

The datasets used and/or analyzed during the current study are available from the corresponding author on reasonable request.

## Abbreviations

BAs: Bile acids
BW: Body weight
CDCA: Chenodeoxycholic acid
CLDN1: Claudin-1
DAO: Diamine oxidase
DCA: Deoxycholic acid
D-LA: D-Lactic acid
FCR: Feed conversion ratio
FI: Feed intake
GCs: Goblet cells
HCA: Hyocholic acid
HDCA: Hyodeoxycholic acid
IL1b: Interleukin 1 beta
IL6: Interleukin 6
IL8: Interleukin 8
JAM2: Junctional adhesion molecule 2
LCA: Lithocholic acid
MUC2: Mucin 2
MYD88: Myeloid differentiation factor 88
PBAs: Primary bile acids
SBAs: Secondary bile acids
TLR4: Toll-like receptor 4
TLR5: Toll-like receptor 5
WG: Weight gain
ZO1: Zonula occludens 1
