# Supplementary material for: Dietary bile acids supplementation protects against Salmonella Typhimurium infection via improving intestinal mucosal barrier and gut microbiota composition in broilers
Source: J Anim Sci Biotechnol. 2024 Nov 12;15:155. doi: 10.1186/s40104-024-01113-5 (PMC11555931; doi:10.1186/s40104-024-01113-5)
Supplement: Supplementary file 1 — Additional file 1. Growth performance and the weight of organs of broilers. [file 40104_2024_1113_MOESM1_ESM.docx]

**Additional file 1** Growth performance and the weight of organs of broilers

| Items | CON | ST | ST-ANT | ST-BA |
| --- | --- | --- | --- | --- |
| Growth performance | | | | |
| 1 d BW, g | 44.53 ± 0.13 | 44.75 ± 0.07 | 44.37 ± 0.05 | 44.70 ± 0.09 |
| 19 d BW, g | 624.40 ± 21.59^a^ | 457.58 ± 17.09^c^ | 540.47 ± 18.83^b^ | 527.46 ± 18.46^b^ |
| 1–19 d FI, g | 709.94 ± 21.62^ab^ | 687.21 ± 13.74^b^ | 701.88 ± 20.27^ab^ | 770.37 ± 23.03^a^ |
| 1–19 d FCR, g/g | 1.15 ± 0.03^c^ | 1.52 ± 0.01^a^ | 1.30 ± 0.06^bc^ | 1.47 ± 0.05^ab^ |
| Organ weight | | | | |
| Liver weight, g | 21.15 ± 0.97^b^ | 23.21 ± 1.47^ab^ | 28.07 ± 1.77^a^ | 24.39 ± 1.74^ab^ |
| Spleen weight, g | 0.73 ± 0.06^b^ | 0.90 ± 0.08^ab^ | 0.84 ± 0.08^ab^ | 1.01 ± 0.06^a^ |
| Heart weight, g | 4.50 ± 0.13^ab^ | 3.95 ± 0.16^b^ | 5.01 ± 0.23^a^ | 4.59 ± 0.22^ab^ |
| Organ indices | | | | |
| Liver index, g/kg | 31.99 ± 1.32^b^ | 52.90 ± 3.47^a^ | 53.97 ± 3.70^a^ | 40.21 ± 3.28^b^ |
| Spleen index, g/kg | 1.09 ± 0.08^b^ | 2.03 ± 0.17^a^ | 1.45 ± 0.11^b^ | 1.62 ± 0.16^ab^ |
| Heart index, g/kg | 6.83 ± 0.22^b^ | 9.29 ± 0.42^a^ | 9.13 ± 0.57^a^ | 7.33 ± 0.25^b^ |

Data are presented as the mean ± SEM (*n* = 8)

^a–c^Different lowercase letters indicate that changes between groups are statistically significant
